# l-Asparagine–l-tartaric acid (1/1)

**DOI:** 10.1107/S1600536810030771

**Published:** 2010-08-11

**Authors:** S. Natarajan, V. Hema, J. Kalyana Sundar, J. Suresh, P. L. Nilantha Lakshman

**Affiliations:** a Department of Physics, Madurai Kamaraj University, Madurai 625 021, India; b Department of Physics, The Madura College, Madurai 625 011, India; c Department of Food Science and Technology, University of Ruhuna, Mapalana, Kamburupitiya (81100), Sri Lanka

## Abstract

In the title compound, C_4_H_8_N_2_O_3_·C_4_H_6_O_6_, the amino acid mol­ecule exists as a zwitterion and the carb­oxy­lic acid in an un-ionized state. The tartaric acid mol­ecules are linked into layers parallel to the *ab* plane by O—H⋯O hydrogen bonds. The amino acid mol­ecules are also linked into layers parallel to the *ab* plane by N—H⋯O and C—H⋯O hydrogen bonds. The alternating tartaric acid and amino acid layers are linked into a three-dimensional framework by N—H⋯O and O—H⋯O hydrogen bonds.

## Related literature

Our inter­est in the determination of the structure of the title compound is due to recent advances in organic non-linear optical (NLO) materials on account of their widespread potential industrial applications. For studies on organic non-linear optical materials, see: Cole *et al.* (2000 ▶); Ravi *et al.* (1998 ▶); Sarma *et al.* (1997 ▶).
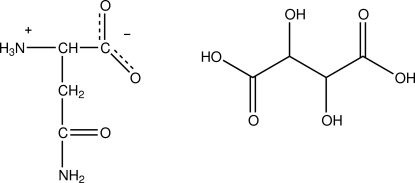


## Experimental

### 

#### Crystal data

C_4_H_8_N_2_O_3_·C_4_H_6_O_6_
*M* *_r_* = 282.21
Monoclinic, 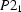

*a* = 5.0860 (4) Å
*b* = 9.6720 (6) Å
*c* = 11.8340 (8) Å
β = 95.311 (8)°
*V* = 579.64 (7) Å^3^
*Z* = 2
Mo *K*α radiation
μ = 0.15 mm^−1^
*T* = 293 K
0.28 × 0.23 × 0.21 mm

#### Data collection

Nonius MACH-3 diffractometer
Absorption correction: ψ scan (North *et al.*, 1968 ▶) *T*_min_ = 0.959, *T*_max_ = 0.969
1339 measured reflections
1073 independent reflections
1015 reflections with *I* > 2σ(*I*)
*R*_int_ = 0.095
2 standard reflections every 60 min  intensity decay: none

#### Refinement

*R*[*F* ^2^ > 2σ(*F* ^2^)] = 0.053
*wR*(*F* ^2^) = 0.226
*S* = 1.38
1073 reflections
172 parameters
1 restraint
H-atom parameters constrained
Δρ_max_ = 0.34 e Å^−3^
Δρ_min_ = −0.40 e Å^−3^

### 

Data collection: *CAD-4 EXPRESS* (Enraf–Nonius, 1994 ▶); cell refinement: *CAD-4 EXPRESS*; data reduction: *XCAD4* (Harms & Wocadlo, 1996 ▶); program(s) used to solve structure: *SHELXS97* (Sheldrick, 2008 ▶); program(s) used to refine structure: *SHELXL97* (Sheldrick, 2008 ▶); molecular graphics: *PLATON* (Spek, 2009 ▶); software used to prepare material for publication: *SHELXL97*.

## Supplementary Material

Crystal structure: contains datablocks global, I. DOI: 10.1107/S1600536810030771/ci5134sup1.cif

Structure factors: contains datablocks I. DOI: 10.1107/S1600536810030771/ci5134Isup2.hkl

Additional supplementary materials:  crystallographic information; 3D view; checkCIF report

## Figures and Tables

**Table 1:** Hydrogen-bond geometry (Å, °)

*D*—H⋯*A*	*D*—H	H⋯*A*	*D*⋯*A*	*D*—H⋯*A*
N1—H1*A*⋯O5^i^	0.86	2.23	3.053 (8)	160
N1—H1*B*⋯O3^ii^	0.86	2.04	2.856 (9)	159
N2—H2*A*⋯O8^iii^	0.89	2.19	2.895 (8)	136
N2—H2*B*⋯O2^iv^	0.89	2.28	2.921 (9)	129
N2—H2*C*⋯O7	0.89	2.06	2.912 (8)	160
N2—H2*C*⋯O8	0.89	2.30	2.916 (8)	126
O4—H4⋯O1^v^	0.82	1.69	2.500 (6)	168
O6—H6⋯O4^vi^	0.82	2.19	2.959 (8)	156
O7—H7⋯O6^iii^	0.82	2.05	2.850 (7)	166
O9—H9⋯O2^vii^	0.82	1.75	2.570 (7)	180
C2—H2⋯O2^iii^	0.98	2.56	3.426 (9)	147
C3—H3*A*⋯O3^ii^	0.97	2.40	3.158 (10)	134

Symmetry codes: (i) 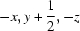; (ii) 
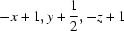
; (iii) 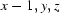; (iv) 
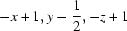
; (v) 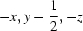; (vi) 
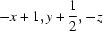
; (vii) 
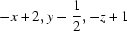
.

**Fig. 1.**
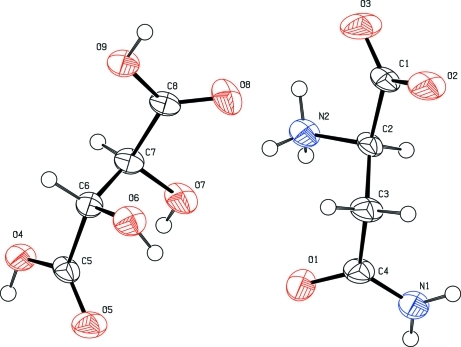
The molecular structure of (I), showing 50% probability displacement ellipsoids and the atom-numbering scheme.

**Fig. 2.**
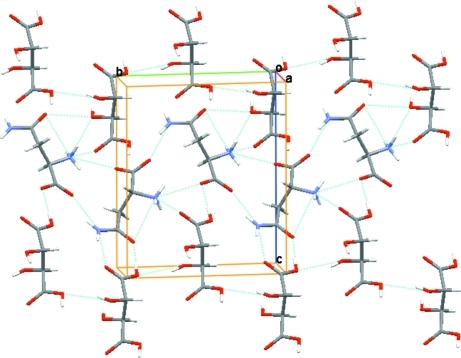
Part of the crystal packing of (I), viewed down the a axis. Hydrogen bonds are shown as dashed lines.

## supplementary crystallographic information

### Comment

Amino acids and carboxylic acids form proton-transfer complexes and hence the ionization states and stoichiometry of individual molecules and their effect on aggregation patterns are of immense interest. Our interest in the determination of the structure of the title compound is due to recent advances in organic non-linear optical (NLO) materials on account of their widespread potential industrial applications. Results have shown that an inherent relationship exists between the structure of these materials and their observed properties. On the molecular scale, the extent of charge transfer is assumed to dominate the SHG output while on the supramolecular scale, a high SHG output requires non-centrosymmetry, strong intermolecular interactions and good phase-matching ability (Sarma *et al.*, 1997: Ravi *et al.*, 1998: Cole *et al.*, 2000)

Fig.1 illustrates the molecular structure of the title compound, (I), and the atomic numbering scheme adopted. The amino acid molecule exists as a zwitterion, an uncommon ionization state in the crystal structures of amino-carboxylic acid complexes. Usually, a proton transfer is favoured from the carboxylic acid to the amino acid in these complexes, the former exists in the anionic state and the latter in the cationic state. Similar zwitterionic state for the amino acid molecule is observed in L-phenylalanine fumaric acid and L-phenylalanine benzoic acid. The asparagine carboxylate skeleton, which includes O2, O3, C1 and C2 is nearly planar. The deviation of the amine N atom from the plane of the carboxylate group is 0.516 (2) Å. The twist of the carboxylate group of the asparagine molecule is described by ψ^1^ = 160.4 (6)° and ψ^2^ = -24.9 (9)°, corresponding to *trans* and *cis* arrangements. The side-chain conformations are observed as χ^1^ = 63.9 (8)°, χ^21^ = -80.7 (9)° and χ^22^ = 95.9 (8)° for the asparagine molecule.

The tartaric acid molecule is in the unionized state. The angle between the planes of the half molecules, O9/O8/C8/C7/O7 and O4/O5/C5/C6/O6 is 57.6 (3)°, which is closer to the value of 54.6° found in the structure of tartaric acid. The carbon skeleton of the tartrate molecule is non-planar, with a C5—C6—C7—C8 torsion angle of - 168.5 (6)°.

Fig. 2 shows the partial packing diagram in which there are large number of O—H···O and N—H···O hydrogen bonds. The tartaric acid molecules are linked into layers parallel to the *ab* plane by O—H···O hydrogen bonds. The amino acid molecules are linked into chains propagating along the *b* axis by N—H···O hydrogen bonds. The chains are arranged in layers parallel to the *ab* plane. The alternating tartaric acid and amino acid layers are linked into a three-dimensional framework by N—H···O and O—H···O hydrogen bonds. In addition, C—H···O hydrogen bonds are observed.

### Experimental

Colourless, prismatic single crystals of (I) were grown from a saturated solution of water containing L-asparagine and tartaric acid in a 1:1 stoichiometric ratio.

### Refinement

In the absence of significant anomalous scattering effects, Friedel pairs were averaged. The absolute configuration was assigned based on the known configuration of L-arginine and L-tartaric acid. The H atoms were placed at calculated positions [O–H = 0.82 Å, N–H = 0.86 or 0.89 Å and C–H = 0.98 Å] and were allowed to ride on their respective parent atoms with U_iso_(H) = 1.2U_eq_(C,N) and 1.5U_eq_(O).

### Crystal data

**Table:**

C_4_H_8_N_2_O_3_·C_4_H_6_O_6_	*F*(000) = 296
*M**_r_* = 282.21	*D*_x_ = 1.617 Mg m^−^^3^
Monoclinic, *P*2_1_	Mo *K*α radiation, λ = 0.71073 Å
Hall symbol: P 2yb	Cell parameters from 25 reflections
*a* = 5.0860 (4) Å	θ = 2.7–25°
*b* = 9.6720 (6) Å	µ = 0.15 mm^−^^1^
*c* = 11.8340 (8) Å	*T* = 293 K
β = 95.311 (8)°	Block, colourless
*V* = 579.64 (7) Å^3^	0.28 × 0.23 × 0.21 mm
*Z* = 2	

### Data collection

**Table:**

Nonius MACH-3 diffractometer	1015 reflections with *I* > 2σ(*I*)
Radiation source: fine-focus sealed tube	*R*_int_ = 0.095
graphite	θ_max_ = 25.0°, θ_min_ = 2.7°
ω–2θ scans	*h* = 0→6
Absorption correction: ψ scan (North *et al.*, 1968)	*k* = −1→11
*T*_min_ = 0.959, *T*_max_ = 0.969	*l* = −14→13
1339 measured reflections	2 standard reflections every 60 min
1073 independent reflections	intensity decay: none

### Refinement

**Table:**

Refinement on *F*^2^	Primary atom site location: structure-invariant direct methods
Least-squares matrix: full	Secondary atom site location: difference Fourier map
*R*[*F*^2^ > 2σ(*F*^2^)] = 0.053	Hydrogen site location: inferred from neighbouring sites
*wR*(*F*^2^) = 0.226	H-atom parameters constrained
*S* = 1.38	*w* = 1/[σ^2^(*F*_o_^2^) + (0.1143*P*)^2^ + 0.7291*P*] where *P* = (*F*_o_^2^ + 2*F*_c_^2^)/3
1073 reflections	(Δ/σ)_max_ = 0.001
172 parameters	Δρ_max_ = 0.34 e Å^−^^3^
1 restraint	Δρ_min_ = −0.40 e Å^−^^3^

### Special details

**Table:**

Geometry. All e.s.d.'s (except the e.s.d. in the dihedral angle between two l.s. planes) are estimated using the full covariance matrix. The cell e.s.d.'s are taken into account individually in the estimation of e.s.d.'s in distances, angles and torsion angles; correlations between e.s.d.'s in cell parameters are only used when they are defined by crystal symmetry. An approximate (isotropic) treatment of cell e.s.d.'s is used for estimating e.s.d.'s involving l.s. planes.
Refinement. Refinement of *F*^2^ against ALL reflections. The weighted *R*-factor *wR* and goodness of fit *S* are based on *F*^2^, conventional *R*-factors *R* are based on *F*, with *F* set to zero for negative *F*^2^. The threshold expression of *F*^2^ > σ(*F*^2^) is used only for calculating *R*-factors(gt) *etc*. and is not relevant to the choice of reflections for refinement. *R*-factors based on *F*^2^ are statistically about twice as large as those based on *F*, and *R*- factors based on ALL data will be even larger.

### Fractional atomic coordinates and isotropic or equivalent isotropic displacement parameters (Å^2^)

**Table:**

	*x*	*y*	*z*	*U*_iso_*/*U*_eq_	
O1	0.0957 (12)	0.4538 (7)	0.1832 (5)	0.0531 (18)	
O2	0.7732 (11)	0.5170 (7)	0.5295 (5)	0.0459 (16)	
O3	0.5946 (13)	0.3196 (8)	0.5798 (5)	0.0544 (18)	
N1	0.0876 (12)	0.6710 (7)	0.2495 (5)	0.0377 (16)	
H1A	−0.0578	0.6889	0.2091	0.045*	
H1B	0.1622	0.7336	0.2930	0.045*	
N2	0.2460 (11)	0.3066 (7)	0.4019 (5)	0.0322 (14)	
H2A	0.0730	0.3170	0.3847	0.039*	
H2B	0.2741	0.2509	0.4617	0.039*	
H2C	0.3176	0.2698	0.3430	0.039*	
C1	0.6013 (12)	0.4214 (8)	0.5193 (5)	0.0280 (15)	
C2	0.3687 (13)	0.4442 (8)	0.4293 (5)	0.0263 (15)	
H2	0.2378	0.5013	0.4633	0.032*	
C3	0.4415 (14)	0.5175 (8)	0.3216 (6)	0.0333 (17)	
H3A	0.5336	0.6030	0.3420	0.040*	
H3B	0.5585	0.4592	0.2822	0.040*	
C4	0.1954 (14)	0.5483 (8)	0.2447 (6)	0.0328 (16)	
O4	0.2756 (10)	−0.0470 (6)	−0.0292 (4)	0.0378 (13)	
H4	0.1664	−0.0392	−0.0843	0.057*	
O5	0.4110 (10)	0.1599 (6)	−0.0849 (5)	0.0396 (14)	
O6	0.8377 (9)	0.1558 (6)	0.0676 (4)	0.0355 (13)	
H6	0.7635	0.2283	0.0478	0.053*	
O7	0.3467 (9)	0.1735 (7)	0.1895 (4)	0.0385 (14)	
H7	0.2136	0.1605	0.1462	0.058*	
O8	0.7364 (9)	0.1781 (7)	0.3463 (5)	0.0418 (15)	
O9	0.9010 (10)	−0.0231 (6)	0.2943 (5)	0.0378 (14)	
H9	1.0049	−0.0102	0.3505	0.057*	
C5	0.4314 (12)	0.0606 (9)	−0.0232 (5)	0.0283 (15)	
C6	0.6460 (13)	0.0500 (8)	0.0750 (6)	0.0269 (15)	
H6A	0.7333	−0.0400	0.0712	0.032*	
C7	0.5159 (12)	0.0584 (8)	0.1865 (6)	0.0296 (15)	
H7A	0.4173	−0.0268	0.1977	0.035*	
C8	0.7309 (12)	0.0778 (8)	0.2842 (5)	0.0274 (16)	

### Atomic displacement parameters (Å^2^)

**Table:**

	*U*^11^	*U*^22^	*U*^33^	*U*^12^	*U*^13^	*U*^23^
O1	0.054 (4)	0.034 (3)	0.061 (4)	0.011 (3)	−0.043 (3)	−0.010 (3)
O2	0.034 (3)	0.052 (4)	0.047 (3)	−0.006 (3)	−0.025 (2)	0.006 (3)
O3	0.052 (4)	0.050 (4)	0.054 (4)	−0.010 (3)	−0.032 (3)	0.018 (3)
N1	0.034 (3)	0.037 (4)	0.039 (3)	0.002 (3)	−0.017 (3)	−0.002 (3)
N2	0.022 (3)	0.039 (4)	0.033 (3)	−0.003 (3)	−0.009 (2)	−0.001 (3)
C1	0.017 (3)	0.042 (4)	0.023 (3)	0.003 (3)	−0.008 (2)	−0.003 (3)
C2	0.023 (3)	0.033 (4)	0.021 (3)	0.003 (3)	−0.008 (3)	−0.004 (3)
C3	0.027 (3)	0.041 (4)	0.030 (3)	−0.003 (3)	−0.010 (3)	0.005 (3)
C4	0.029 (3)	0.036 (4)	0.031 (3)	−0.001 (3)	−0.011 (3)	0.003 (3)
O4	0.035 (3)	0.033 (3)	0.041 (3)	−0.005 (3)	−0.023 (2)	0.001 (3)
O5	0.034 (3)	0.043 (3)	0.038 (3)	−0.001 (3)	−0.012 (2)	0.008 (3)
O6	0.023 (2)	0.041 (3)	0.040 (3)	−0.004 (2)	−0.0068 (19)	0.001 (3)
O7	0.021 (2)	0.054 (4)	0.037 (3)	0.008 (2)	−0.0123 (19)	−0.008 (3)
O8	0.023 (2)	0.056 (4)	0.044 (3)	0.004 (3)	−0.015 (2)	−0.019 (3)
O9	0.029 (3)	0.041 (3)	0.039 (3)	0.006 (2)	−0.019 (2)	0.000 (3)
C5	0.021 (3)	0.037 (4)	0.026 (3)	0.006 (3)	−0.007 (2)	−0.006 (3)
C6	0.019 (3)	0.029 (3)	0.030 (4)	0.001 (3)	−0.008 (3)	−0.002 (3)
C7	0.019 (3)	0.036 (4)	0.031 (3)	0.002 (3)	−0.011 (2)	−0.001 (3)
C8	0.017 (3)	0.039 (4)	0.025 (3)	−0.002 (3)	−0.005 (2)	0.003 (3)

### Geometric parameters (Å, °)

**Table:**

O1—C4	1.247 (10)	O4—C5	1.306 (10)
O2—C1	1.271 (10)	O4—H4	0.82
O3—C1	1.219 (10)	O5—C5	1.205 (10)
N1—C4	1.311 (11)	O6—C6	1.422 (9)
N1—H1A	0.86	O6—H6	0.82
N1—H1B	0.86	O7—C7	1.409 (9)
N2—C2	1.492 (10)	O7—H7	0.82
N2—H2A	0.89	O8—C8	1.215 (9)
N2—H2B	0.89	O9—C8	1.302 (9)
N2—H2C	0.89	O9—H9	0.82
C1—C2	1.533 (9)	C5—C6	1.522 (9)
C2—C3	1.533 (10)	C6—C7	1.532 (10)
C2—H2	0.98	C6—H6A	0.98
C3—C4	1.507 (9)	C7—C8	1.528 (8)
C3—H3A	0.97	C7—H7A	0.98
C3—H3B	0.97		
			
C4—N1—H1A	120.0	O1—C4—C3	118.6 (7)
C4—N1—H1B	120.0	N1—C4—C3	118.7 (7)
H1A—N1—H1B	120.0	C5—O4—H4	109.5
C2—N2—H2A	109.5	C6—O6—H6	109.5
C2—N2—H2B	109.5	C7—O7—H7	109.5
H2A—N2—H2B	109.5	C8—O9—H9	109.5
C2—N2—H2C	109.5	O5—C5—O4	125.7 (6)
H2A—N2—H2C	109.5	O5—C5—C6	122.1 (7)
H2B—N2—H2C	109.5	O4—C5—C6	112.1 (6)
O3—C1—O2	126.0 (6)	O6—C6—C5	110.6 (6)
O3—C1—C2	117.5 (6)	O6—C6—C7	111.6 (6)
O2—C1—C2	116.2 (7)	C5—C6—C7	108.5 (5)
N2—C2—C3	111.1 (5)	O6—C6—H6A	108.7
N2—C2—C1	107.6 (6)	C5—C6—H6A	108.7
C3—C2—C1	114.5 (5)	C7—C6—H6A	108.7
N2—C2—H2	107.8	O7—C7—C8	106.3 (6)
C3—C2—H2	107.8	O7—C7—C6	112.2 (6)
C1—C2—H2	107.8	C8—C7—C6	108.8 (5)
C4—C3—C2	110.0 (6)	O7—C7—H7A	109.8
C4—C3—H3A	109.7	C8—C7—H7A	109.8
C2—C3—H3A	109.7	C6—C7—H7A	109.8
C4—C3—H3B	109.7	O8—C8—O9	124.4 (6)
C2—C3—H3B	109.7	O8—C8—C7	122.0 (6)
H3A—C3—H3B	108.2	O9—C8—C7	113.6 (6)
O1—C4—N1	122.6 (6)		
			
O3—C1—C2—N2	−24.9 (9)	O5—C5—C6—C7	111.3 (8)
O2—C1—C2—N2	160.4 (6)	O4—C5—C6—C7	−67.4 (8)
O3—C1—C2—C3	−149.0 (7)	O6—C6—C7—O7	70.9 (7)
O2—C1—C2—C3	36.4 (9)	C5—C6—C7—O7	−51.2 (8)
N2—C2—C3—C4	63.9 (8)	O6—C6—C7—C8	−46.4 (8)
C1—C2—C3—C4	−173.9 (6)	C5—C6—C7—C8	−168.5 (6)
C2—C3—C4—O1	−80.7 (9)	O7—C7—C8—O8	−0.7 (9)
C2—C3—C4—N1	95.9 (8)	C6—C7—C8—O8	120.3 (7)
O5—C5—C6—O6	−11.4 (9)	O7—C7—C8—O9	178.5 (6)
O4—C5—C6—O6	170.0 (6)	C6—C7—C8—O9	−60.6 (8)

### Hydrogen-bond geometry (Å, °)

**Table:**

*D*—H···*A*	*D*—H	H···*A*	*D*···*A*	*D*—H···*A*
N1—H1A···O5^i^	0.86	2.23	3.053 (8)	160
N1—H1B···O3^ii^	0.86	2.04	2.856 (9)	159
N2—H2A···O8^iii^	0.89	2.19	2.895 (8)	136
N2—H2B···O2^iv^	0.89	2.28	2.921 (9)	129
N2—H2C···O7	0.89	2.06	2.912 (8)	160
N2—H2C···O8	0.89	2.30	2.916 (8)	126
O4—H4···O1^v^	0.82	1.69	2.500 (6)	168
O6—H6···O4^vi^	0.82	2.19	2.959 (8)	156
O7—H7···O6^iii^	0.82	2.05	2.850 (7)	166
O9—H9···O2^vii^	0.82	1.75	2.570 (7)	180
C2—H2···O2^iii^	0.98	2.56	3.426 (9)	147
C3—H3A···O3^ii^	0.97	2.40	3.158 (10)	134

Symmetry codes: (i) −*x*, *y*+1/2, −*z*; (ii) −*x*+1, *y*+1/2, −*z*+1; (iii) *x*−1, *y*, *z*; (iv) −*x*+1, *y*−1/2, −*z*+1; (v) −*x*, *y*−1/2, −*z*; (vi) −*x*+1, *y*+1/2, −*z*; (vii) −*x*+2, *y*−1/2, −*z*+1.
